# Terracotta: A tool for conducting experimental research on student learning

**DOI:** 10.3758/s13428-023-02164-8

**Published:** 2023-07-10

**Authors:** Benjamin A. Motz, Öykü Üner, Harmony E. Jankowski, Marcus A. Christie, Kim Burgas, Diego del Blanco Orobitg, Mark A. McDaniel

**Affiliations:** 1 Department of Psychological and Brain Sciences, Indiana University Bloomington, 1101 East 10th Street, Bloomington, IN 47405 USA; 2 Pervasive Technology Institute, Indiana University, Bloomington, IN 47405 USA; 3 New York, USA; 4 Unicon, Inc., Gilbert, AZ USA; 5 Department of Psychological and Brain Sciences, Washington University in St. Louis, St. Louis, MO USA

**Keywords:** Experiments, Education, Student learning, Learning management system (LMS), Retrieval practice

## Abstract

For researchers seeking to improve education, a common goal is to identify teaching practices that have causal benefits in classroom settings. To test whether an instructional practice exerts a causal influence on an outcome measure, the most straightforward and compelling method is to conduct an experiment. While experimentation is common in laboratory studies of learning, experimentation is increasingly rare in classroom settings, and to date, researchers have argued it is prohibitively expensive and difficult to conduct experiments on education in situ. To address this challenge, we present Terracotta (Tool for Education Research with RAndomized COnTrolled TriAls), an open-source web application that integrates with a learning management system to provide a comprehensive experimental research platform within an online class site. Terracotta automates randomization, informed consent, experimental manipulation of different versions of learning activities, and export of de-identified research data. Here we describe these features, and the results of a live classroom demonstration study using Terracotta, a preregistered replication of McDaniel et al. (*Journal of Applied Research in Memory and Cognition, 1*(1), 18–26, 2012). Using Terracotta, we experimentally manipulated online review assignments so that consenting students alternated, on a weekly basis, between taking multiple-choice quizzes (retrieval practice) and reading answers to these quizzes (restudy). Students' performance on subsequent exams was significantly improved for items that had been in retrieval practice review assignments. This successful replication demonstrates that Terracotta can be used to experimentally manipulate consequential aspects of students’ experiences in education settings.

## Introduction

The productivity and resilience of society depends on our system of formal education, and this system currently suffers major challenges, including lagging achievement, low persistence in science, technology, engineering, and math (STEM) disciplines, and systematic inequities based on students' sociodemographic characteristics. These challenges are well documented across a range of national and international assessment instruments (e.g., NAEP, 2022; NCES, 2022; PISA, 2020). However, despite having strong instruments for measuring these issues, there has been a complete lack of tools for research on how these issues should be remedied.

Improving education involves identifying and promoting instructional practices that have causal benefits for student outcomes (National Science Foundation & Institute for Education Sciences, 2013; US Department of Education, 2016, 2017). To test whether an instructional practice exerts a causal influence on an outcome measure, the most straightforward and compelling research method is to conduct an experiment (National Research Council, 2002; Whitehurst, 2003), and in particular, to embed this experiment *in an education setting*, yielding causal inferences that are authentic to the contexts where they matter in practice (Koedinger et al., 2013; Motz et al., 2018). An experiment satisfies the strong requirements of causal inference by providing evidence that a change in behavior is attributable to a change in treatment, in a specific direction (ruling out reverse causality), and by minimizing (through random assignment) the possibility that it is explainable through other causal mechanisms. Combined, these features constitute an ideal method for examining whether a treatment (e.g., a learning activity, an instructional strategy, a motivational intervention) causes improvements (Mosteller & Boruch, 2002; Shadish et al., 2001). Unfortunately, however, conventional education settings are not naturally conducive to experimentation.

Researchers have repeatedly argued that it is prohibitively difficult to conduct controlled experiments in education settings. Slavin (2002) notes that “randomized experiments of interventions applying to entire classrooms can be extremely difficult and expensive to do” (p. 17). Similarly, Sullivan (2011) comments on the “feasibility considerations” of conducting experiments in education, remarking that the cost “can be high and industry support for education trials rarely exists” (p. 285). Such concerns are also expressed by Levin (2005), who writes, “the requisite resources are generally far in excess of what most educational researchers could hope to amass in the absence of considerable extramural funding. Consequently, researchers elect to conduct more manageable, less ambitious, and typically, less carefully-controlled classroom-based investigations” (p. 19). Perhaps for these reasons, the relative frequency of experimental educational research studies has been in steady decline, from 47% of published education studies in 1983, to 40% in 1994, to 26% in 2004, to 20% in 2020, despite increasing prevalence of causal claims in the education research literature (Brady et al., 2022; Hsieh et al., 2005; Motz et al., 2023; Robinson et al., 2007).

Experimental psychologists have been filling this gap to some extent, conducting experimental studies on human learning, albeit primarily under controlled laboratory conditions, and then advocating for their applicability in education settings (Benassi et al., 2014; Pashler et al., 2007; Roediger & Pyc, 2012). Their advocacy, however, has been restrained; these same experimental psychologists, and others as well, affirm that research is needed to validate claims in practice (Daniel, 2012; Koedinger et al., 2013; Motz et al., 2018). For example, in Dunlosky et al.'s (2013) extensive review of learning strategies from cognitive and educational psychology, the evidence from education settings is marked as “insufficient” for 8 out of the 10 strategies under investigation. This evidence is important for testing the generalizability of effects within the complexities of education settings (de Leeuw et al., 2022; Fyfe et al., 2021) and to build an evidence base that is more convincing and relevant to practitioners. However, despite advocating for the importance of this translational research, experimental and educational psychology have offered no fix for the methodological difficulties that have impeded experimentation in education for the past 40 years.

To be fair, experimental research in authentic, practical settings is difficult in *all* disciplines, and some researchers manage through it; the difficulties of experiments in education are not insurmountable, provided some struggle (Gueron, 2002). But a problematic feature of experimental research on student learning in practice, specifically, has been the complete lack of *tools* to support it (Schneider & Garg, 2020). This absence of experimental research infrastructure has been particularly vexing when considering that improving education is a critical research priority: all members of society are directly affected by our education system, and by its challenges.

Fortunately, things are starting to change. Enabled by the digital transformation of education, research tools for experimentally manipulating features of the student learning environment have begun to emerge (Baker et al., 2022; McCarthy et al., 2022). As student learning activities and student data are increasingly online, the technical infrastructure for supporting education can be leveraged for research purposes—digital learning platforms can support student learning, *and also* can be equipped with tools to support experimental research on education. In this article, we describe one such emerging tool, Terracotta, and we present a demonstration of Terracotta's capabilities in a class-embedded preregistered replication experiment.

### Overview of Terracotta

Terracotta (Tool for Education Research with RAndomized COnTrolled TriAls; https://terracotta.education) is an online experiment builder that allows researchers and teachers to rapidly design and deploy experiments directly within the learning management system (LMS). The LMS is now a prominent and integral component of formal education settings, central to routine practice akin to how the chalkboard was once the center of the classroom. It is a large-scale online platform that hosts secure websites for each class within a district or an institution and makes a suite of applications available for student and teacher use. These include organizational frameworks like customizable pages and modules, as well as file and multimedia storage, assignments, quizzes, announcements, calendars, private messaging, discussions, gradebooks, and other functions. Adoption of an LMS has for years been driven by education’s ongoing digital transformation (Lonn & Teasley, 2009; Pomerantz & Brooks, 2017; Staker, 2011), but has accelerated rapidly due to COVID-19, particularly in K–12 environments where LMS adoption more than doubled between 2019 and 2020 (Hill, 2020). By centering in the LMS, Terracotta enables experimental research across education levels, student populations, and learning materials.

Presently, Terracotta's primary feature is to experimentally manipulate LMS assignments. Assignments instruct students to perform learning activities, and as such, assignments are the vehicles that connect students with practice, feedback, resources, interventions, and formative assessments. In many ways, assignments occupy a central element of education systems, because when students have autonomy to choose their own learning strategies, long-standing evidence shows that students’ choices are often suboptimal (Kornell & Bjork, 2007; Pressley et al., 1989). Furthermore, assignments are increasingly used to administer social and motivational interventions in education settings (Harackiewicz & Priniski, 2018; Yeager et al., 2019). Thus, what a teacher should assign, and how they should assign it, represent foundational priorities for education research (Benassi et al., 2014; Borman, 2002; Cohen et al., 2003; Dunlosky et al., 2013). At a high level, Terracotta makes it possible to create different versions of an LMS assignment (which might vary in instructions, contents, resources, etc.) and to randomly assign students to experience these different versions.

**Fig. 1:**
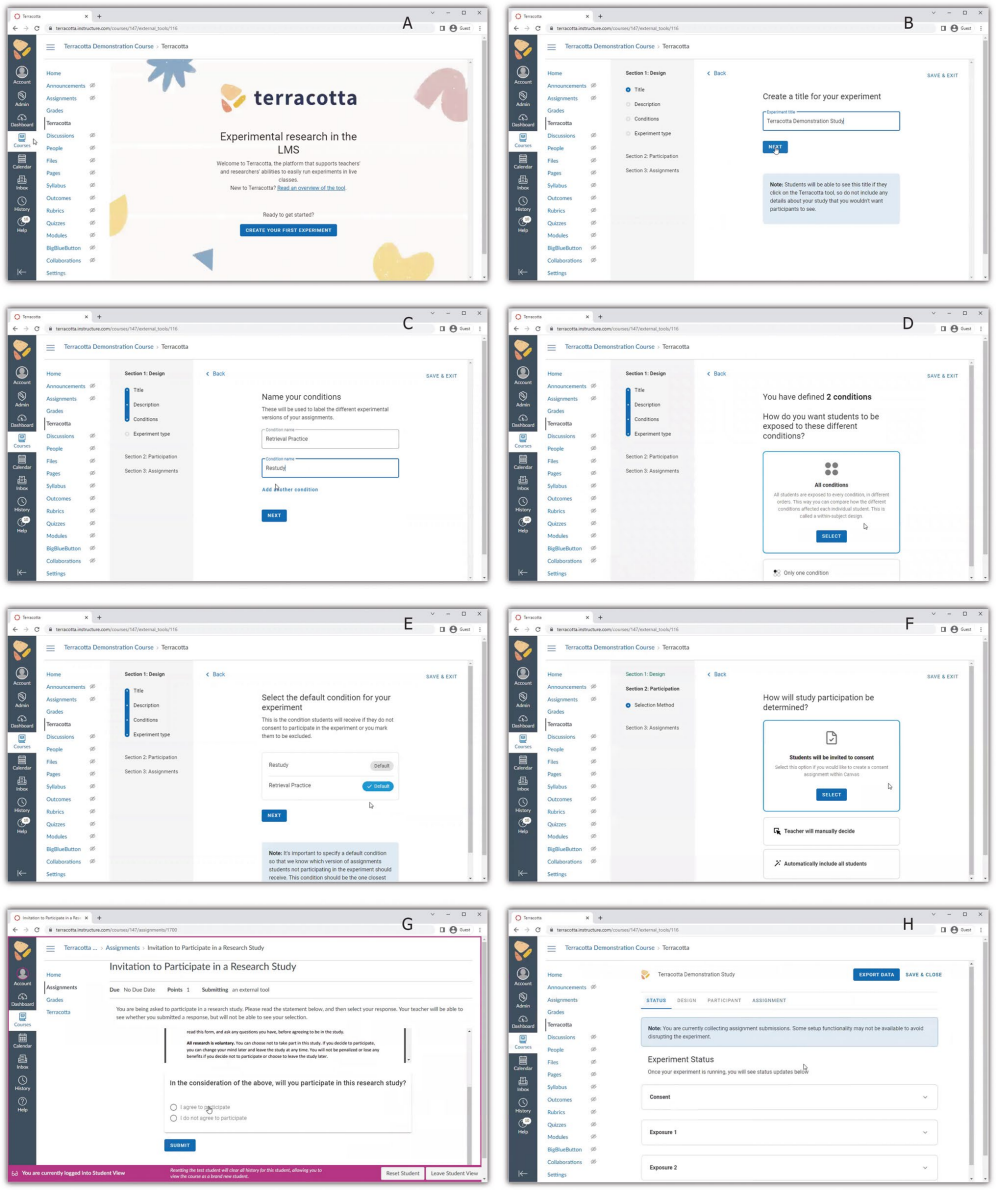
Screenshots of Terracotta, as integrated in the Canvas LMS (Instructure; Salt Lake City, UT). A welcome frame is displayed when a user first accesses Terracotta (panel **A**), and key steps of the Terracotta interactive guide are shown in panels **B**–**F**. The student view of a consent assignment is shown in panel **G**, and the experiment status screen is shown in panel **H**. A video of the demonstration study’s setup is available at https://osf.io/24qp7

An experiment in Terracotta is created using an interactive guide (see screenshots in Fig. 1). Like the US Department of Education’s Evidence-to-Insight (e2i) Coach (formerly RCE Coach; Office of Educational Technology, 2017), Terracotta walks the researcher through a sequence of design decisions, such as the number of treatment conditions, treatment design, informed consent, uploading materials, and so on. But unlike e2i Coach, which then leaves researchers to go out and conduct studies on their own, Terracotta embeds the experiment, as designed, automatically within the LMS. From the student's perspective, they complete assignments in the LMS as usual, but behind the scenes, Terracotta manages the details of presenting the appropriate experimental variations to different students and collecting data along the way.

### Ethics of experimentation in education

At first glance, it may seem ethically questionable to experiment on students in real education settings. But in any classroom, whenever a teacher tries something new, a sort of uncontrolled experiment is taking place, and students are subject to the same risks of potentially inferior treatment as in a controlled experiment. In education, this kind of uncontrolled, small sample “research” is seen as a positive feature of teachers’ professional development and growth (Guskey & Huberman, 1995). However, this “research” (a teacher trying new things on a full student cohort) also has the drawbacks of being subject to the single unit confound (any improvement could be due to other properties of the cohort) and being subject to bias (whether new tactics “work” is only judged by subjective reflection of the teacher, who is also the creator of these tactics). Researchers can avoid these issues by randomly assigning students to experimental conditions. When doing so, it becomes possible to conduct more robust assessments of these instructional treatments, and to advance improvements that are known to benefit students.

Nevertheless, this kind of research is sensitive. When technologies unilaterally enroll their users in experiments, it has been met with strong public objection (Goel, 2014; Herold, 2018). The objection is not simply that experimental research is being performed; instead, such instances have revealed that people expect to be informed when they might be participating in research studies, and they want to be able to decide whether or not they will participate, even if such expectations are not codified in law or policy (Flick, 2015). What *is* supported by law, however, is the expectation that identifiable student records and research data must remain private. Terracotta’s features are intended to achieve these normative expectations directly and systematically.

We believe that the benefits of transparency and agency generally outweigh any challenges that they may present to an embedded research study. In this regard, we advocate that students (and parents, in the case of research on minors) should be informed about an experimental research study that is occurring in Terracotta, and that Terracotta's informed consent feature should be used to provide them with a secure and private means for registering their choice of whether to participate. In situations when permission from a parent or guardian is required, it is possible to distribute permission forms, and then to manually mark in Terracotta which students have returned these forms (or conversely, to mark which students' parents have opted out). The concern that students' knowledge of being in an experiment may change their behaviors, sometimes called the Hawthorne effect, has a shaky evidence base in education research (Adair, 1984). It is, however, possible that individuals from historically oppressed populations may be less likely to agree to participate (e.g., Li et al., 2022), which may yield a biased sample. These issues might best be remedied with *more* transparency during recruitment (Yancey et al., 2006), rather than forgoing consent to reduce sampling bias.

Another ethical concern is that an experiment may cause some students to receive inferior treatment. Simply by differentiating students' learning experiences, it stands to reason that one experimental variant might be better than or worse than another. To proactively mitigate this risk, and its potential effects, we recommend a variety of strategies (Motz et al., 2018). First, an experimental condition that is known to be inferior, such as a deprived or no-treatment control, should not be administered to students; instead, we recommend making comparisons with a *business-as-usual* control (Willingham & Daniel, 2021). Second, whenever possible, we recommend adopting a within-subjects (or delayed treatment) design. In doing so, all participants will experience all conditions, staggered in time, thus equating treatment between students overall. Third, we recommend that the scope of an embedded experiment should be modest—in research (as in routine instruction), large changes to a student's curricular experience may be inappropriate if the benefit of the change is unknown. When keeping an experiment’s scope modest, if experimental differences are observed, they may nevertheless be negligible for any individual student's overall learning and achievement. Fourth, instructional assistance should never be withheld from students. If a student asks a question that is relevant to an experiment, it is more important for the student to receive help than for the study's strictures of experimental control to be maintained. To help ensure that students are treated equitably, Terracotta does not reveal to the teacher the condition to which a student is assigned^1^.

Finally, we affirm that individual students' identities, their decisions to participate in a study (or not), their behaviors within Terracotta, and their learning performance are confidential and private. When data are exported from Terracotta, the data are de-identified (student identifiers are replaced by otherwise meaningless Terracotta-internal IDs), and data from nonparticipants are excluded from the export. Ideally, researchers will have no need for seeing identifiable research data. Furthermore, we recommend that researchers should clarify, both during recruitment and in an informed consent statement, how participant data will be used following the study.

### Terracotta enables collaboration between teachers and researchers

When we tell people about Terracotta, they often ask: *Who is supposed to use it? Teachers or researchers?* The answer is “Both, together.”

As digital tools for education research emerge, a known risk is that they will marginalize the teacher and neglect important details of the educational implementation, instead focusing myopically on the inner workings of the tool itself. Proponents of research within digital learning platforms advocate for greater involvement of teachers in the research process (Baker et al., 2022; McCarthy et al., 2022), as others have advocated more broadly (Joyce & Cartwright, 2019). This is also our goal with Terracotta.

In an LMS course site, as in a classroom, teachers are in control—they have express privileges to create assignments, course policies, resources, announcements, and so on. Historically, researchers who might want to manipulate these features of a class would need to partner, negotiate, and coordinate with teachers (unless the teachers *are* the researchers; Handelsman et al., 2004; Boyer, 1990). Similarly, Terracotta relies on teacher involvement and enables researchers and teachers to collaborate on how an experimental study will be implemented in a class. A researcher who wants to conduct a study in Terracotta will need to partner with a teacher who is willing to embed the study in their class and collaborate on how an experimental contrast might be applied to the teacher's instructional materials. In practice, the teacher might invite the researcher into the class's LMS site to set up an experiment in Terracotta, or a teacher might create the experiment themselves, in collaboration with the researcher, using Terracotta's interactive guide. Terracotta automatically manages the details of informed consent, random assignment, experimental differentiation, data de-identification, and so on; accordingly, there is minimal effort required of the teacher once a study is initially set up in Terracotta. There are reciprocal benefits to such collaborations: this improves the external validity of a research study, while also involving teachers in building a more authentic and relevant evidence base of what works in routine contexts.

### Key features

#### Informed consent that conceals responses from the instructor

Informed consent is a cornerstone in the ethical conduct of research with humans. While consent may not be *legally* required of research with Terracotta (current code provides an exemption for research on normal educational practices; Exempt Research, § 46.104(d)(1), 2018), consent (or assent, in the case of research with minors) nevertheless improves the transparency of research and provides agency to participants. To realize these benefits, Terracotta implements an LMS assignment that presents an informed consent statement followed by a simple consent prompt, enabling students to mark whether they agree to participate in a research study. Like any assignment in Terracotta, this consent assignment can have a deadline, and it can be configured to give students credit for responding. However, there is no correct answer to the consent prompt, and students will receive submission credit regardless of their response. When a student marks their consent, they become a *participant*, eligible to be randomly assigned to experimental conditions and to have their de-identified data included in the experiment's data export. When a student does not provide consent (either by providing a negative response or by not responding at all), they will not become a participant, they will not be randomly assigned to experimental conditions (they will only receive whichever condition the researcher has marked as "default"), and their data will not be included in the experiment's data export.

An important aspect of Terracotta's consent process is that students' consent responses are never revealed to the teacher or the researcher. This is because many review boards are sensitive that a teacher is in a position of power, and teachers might be perceived as coercing students to participate if they were able to see individual students' consent responses. Terracotta does allow teachers to see *whether* a student has responded to the consent assignment, but it conceals any information about *how* students have responded.

#### Assignment of students to experimental conditions

An experiment's fundamental feature is the introduction of different experimental conditions to different subjects. By default in Terracotta, participants are assigned to a condition (in a between-subjects design) or to a schedule of conditions (in a within-subjects design) at the time when they first access an experimentally manipulated activity in Terracotta. We prefer this *trickle assignment* approach (Riecken & Boruch, 1974) rather than batch assignment, because class enrollment is not necessarily static—students may be added to the LMS course site after the start of the academic term, possibly after an experiment is created, and these students should not be automatically ineligible to participate, nor should they have instructional resources withheld. However, it is also possible in Terracotta to manually assign participants to conditions in a single batch during experiment setup, overriding Terracotta's default behavior.

While pure random assignment may be considered the *de facto* method for assigning participants to conditions, Terracotta implements a hybrid random/sequential assignment algorithm that aims to balance the quantity of participants assigned in each condition. When a participant first accesses an experimentally manipulated activity in Terracotta, they are randomly assigned to a condition. When a subsequent participant accesses an activity in Terracotta, they are assigned to whichever condition has the least number of participants; if two or more conditions have the least number of participants, they are randomly assigned among them. If all conditions have the same number of participants, again, inbound participants are randomly assigned among them. The benefit of this approach is that it optimizes balance in the number of participants assigned to different experimental conditions, while still preserving the lack of bias that is characteristic of random assignment. Balance is a priority in class-embedded experiments, where sample size is limited by the class's enrollment, and it is important to actively avoid the rare possibility that pure random assignment will yield disproportionate group sizes. However, in a between-subjects design, if imbalance is desirable, Terracotta allows the researcher to specify a custom distribution scheme for different experimental conditions (e.g., 75% of participants could be assigned to condition A, and 25% to condition B), and the algorithm described previously will aim to achieve these custom proportions. And again, a researcher could override these processes entirely, and could manually assign participants to conditions when setting up the experiment.

#### Repeated treatments and within-subject crossovers

An occasional criticism of embedded experiments in education is that they are short-term manipulations, "limited to testing the impact of pulling a single lever at a time" (Schanzenbach, 2012). Such criticisms are misleading for at least two reasons. First, some specific levers, even in their transience, can be consequential in education (Walton & Cohen, 2011; Yeager et al., 2019), but second and more broadly, it is simply not true that embedded experiments need to be short-term. Terracotta allows an experimental contrast to be applied across many treatments. A researcher could manipulate a single assignment or could manipulate months-long sequences of assignments (as we did in the Demonstration Study, below). Doing so may increase the "dosage" of a treatment regimen, while also providing a more authentic measure of the regimen's effect if it were routinized in normal instructional practice. Moreover, by manipulating multiple treatments, the effect of an experimental manipulation may be claimed to generalize beyond the nuances of any single assignment.

In allowing repeated treatments, Terracotta also supports the ability for participants to change conditions across treatments, in a within-subjects design. A within-subjects design has important statistical advantages and ethical benefits, but it also introduces the risk of carry-over effects, and the decision to use a within-subject design should be made with an awareness of these tradeoffs (Greenwald, 1976). Once assignments have been created in Terracotta, the timing of these assignments (the open and due date) is configured in the LMS, so the schedule of when participants are exposed to each condition is highly customizable. For example, a participant could experience condition A for four assignments, then cross over to experience condition B for four more assignments (AAAABBBB; a single crossover). Alternatively, a participant could alternate between A and B repetitively (ABABABAB; as we did in the Demonstration Study, below), with multiple crossovers.

#### Mapping outcomes to experimental treatments

In routine educational practice, a teacher measures student learning outcomes (e.g., exam scores) and other relevant behavioral outcomes (e.g., attendance, classroom conduct, participation). In an experiment, these might be relevant as pretest or posttest measures. To assess whether an experimental manipulation in Terracotta affects these distal measures, there needs to be a mapping from these distal measures onto students' research profiles. Without Terracotta, mapping outcomes to experimental treatments can be a sensitive task, as it involves joining identifiable data from different sources (research data and student data). To reduce the risk of loss of confidentiality in the research process, Terracotta includes a feature where outcome data can be identified directly in the LMS gradebook following each treatment exposure (for within-subject designs) or at the end of an experiment (for between-subject designs). Alternatively, if the research is targeting outcome measures that are not in the LMS gradebook (or a sub-score of a gradebook item), Terracotta allows outcomes to be manually entered into a simple class roster for the full class, preventing any exposure of participant's consent decisions or condition assignments. De-identified outcome data, whether selected from the LMS gradebook or manually added, are then included in the Terracotta data export, with nonparticipants removed.

#### Export of de-identified data

At the end of the experiment, Terracotta produces an export of all study data, with student identifiers replaced with random codes, and with non-consenting students removed. This export includes condition assignments, responses and scores on manipulated learning activities, granular clickstream data for interactions with Terracotta assignments, and outcomes data as specified by the teacher. By joining these data, de-identifying it, and excluding non-consenting participants, Terracotta prepares a data export that is shareable with research collaborators (includes no personally identifiable information) and that meets ethical requirements (excludes data from nonparticipants).

### Technical description

Terracotta is an open-source web application; the full source code is available at https://github.com/terracotta-education/terracotta under a permissive Apache 2.0 license (Sinclair, 2010). The Terracotta backend architecture uses a model-view-controller (MVC) pattern written in Java using the Spring framework (https://spring.io). The Terracotta frontend is written in JavaScript using the Vue framework (https://vuejs.org).

Terracotta integrates with an LMS using current learning tool interoperability (LTI; version 1.3) standards (1EdTech, 2022a; Unicon Inc., 2019). User authentication takes place within the LMS, and the LMS provides Terracotta with an encrypted LTI token when a user launches Terracotta. This token identifies the user, their role (learner, instructor), and their context (the LMS course number), so that when a user requests a resource within Terracotta (e.g., a student attempts to complete an assignment), Terracotta can respond with the appropriate experimental treatment (or display the teacher interface, if appropriate). However, the LTI standards do not currently provide all the requisite endpoints to support Terracotta's features, so Terracotta also uses the LMS's native application programming interface (API) to make functional requests within the LMS course site that are outside the scope of LTI 1.3. These API calls are made on behalf of the instructor, using an OAuth 2.0 token explicitly granted by the instructor when first accessing Terracotta. While contemporary LMSes have the same general API endpoints, there are modest differences in them, and thus Terracotta is not universally interoperable by default. At this time, Terracotta is designed for integration with the Canvas LMS (Instructure; Salt Lake City, UT), but we anticipate extending support to other LMSes over time.

Timestamps collected by Terracotta (the time when a student provided consent, started an assignment, clicked submit, etc.) are logged on the server side using the thread-safe Java .now() method.

Terracotta’s user interface uses the open-source Material Design system (Google, 2022) so that the user experience is familiar to users who are accustomed to common web interfaces. To benefit the broadest community possible, every frame in Terracotta is screened using the axe DevTools extension to test for common accessibility issues. Terracotta aspires to comply with Web Content Accessibility Guidelines (WCAG) 2.1 AA standards both within the tool, on its website, and in accompanying documentation.

As an open-source web application, one could self-host their own instance of Terracotta on their own infrastructure. However, Indiana University, Terracotta's home institution, hosts a multi-tenant service in the Amazon Web Services (AWS) Cloud, with elastic scaling and industrial-grade security, currently provided free of charge to US-based districts and institutions who are interested in supporting experimental education research.

### Terracotta data and vocabulary

At the conclusion of an experiment in Terracotta, a de-identified data export can be downloaded from the Terracotta web interface as a zipped archive. This archive contains a set of CSV (comma-separated value) files that describe all aspects of the experiment, as well as a JSON (JavaScript object notation) file that contains participants' timestamped interactions with Terracotta as an event stream. The JSON file is formatted according to the Caliper standard (1EdTech, 2022b). However, to our knowledge, there is currently no common data format for representing complex experimental research studies, and thus, the CSVs contained in Terracotta's data export adopt a novel data structure. Some of these elements can be mapped to the Common Education Data Standards (US Department of Education, 2022), and to facilitate this mapping, an alignment tool is provided with Terracotta's data dictionary. Key Terracotta vocabulary is described in Table 1, along with specific examples from our Demonstration Study, below. Many of the concepts in Terracotta's data vocabulary will be familiar to those with experience with LMS assignments and experimental research. However, we introduce one novel concept to organize and articulate the experiment's structure: an *exposure set*.

**Table 1:** Terracotta vocabulary

Term	Definition and example from the Demonstration Study
Assignment	*An assignment in the learning management system*. For example, the Week 8 Review is an assignment (assignment_id = 413).
Condition	*An experimental variation that generalizes across possible assignments*. For example, "Retrieval Practice" and "Restudy" are two conditions (condition_id = 1088 and 1087, respectively).
Treatment	*A specific combination of an assignment and a condition*. For example, the "Retrieval Practice" version of the Week 8 Review is one treatment (treatment_id = 602), and the "Restudy" version of the Week 8 Review is a different treatment (treatment_id = 601).
Participant	*A student who is participating in an experiment in Terracotta*, either because they have provided consent (or assent), or because they have been marked as a participant by the teacher or researcher (e.g., participant_id = 2877).
Exposure set	*The set of assignments in which a participant experiences one condition.* In a between-subject design, there is only one exposure set. In a within-subject design, there are as many exposure sets as conditions. For example, half the participants experienced "Retrieval Practice" in the reviews for Weeks 8, 10, 12, and 14, while the other half of participants experienced "Restudy" for these assignments. This set of assignments constitutes one exposure set (exposure_id = 864).
Submission	*The work a participant provides in response to an assignment*. For example, participant_id 2877 submitted work to the Week 8 Review (assignment_id = 413) four times. Each of these attempts has its own submission_id (1750, 1923, 1937, and 1943).
Item	*An individual question or prompt within a treatment*. For example, the "Retrieval Practice" version of the Week 8 Review (treatment_id = 602) had 10 different questions, and each of these questions has its own item_id (e.g., item_id = 1189).
Response	*A possible response to a multiple-choice item which participants might select*. For example, there are four response options available on item_id 1189, response_id = 2602, 2603, 2604, and 2605. Any response(s) may be marked as either correct or incorrect.
Item response	*The response that was actually selected (in a multiple-choice item) or provided (in a short answer item) by a participant in response to an item in a single submission*. For example, in submission_id 1750, participant_id 2877 selected response_id 2605 in response to item_id 1189. This selection on this item, in this particular submission attempt is item_response_id 7566.
Outcome	*Numeric values, either selected from the LMS gradebook or manually entered into Terracotta, used to quantify a participant's performance relative to an exposure set*. For example, participants' scores on exam questions that were relevant to the Week 8 Review (exposure_id = 864) were entered as outcome_id = 267.

More information is available at https://terracotta.education/help-center/data-dictionary. The complete data export from the Demonstration Study is available at https://osf.io/yrbhe/

An exposure set is a set of assignments in which a participant experiences one condition. In a between-subjects design, there will be only one exposure set, because each participant will only experience one condition throughout the entirety of the study. However, in a within-subjects design, participants will experience the same number of exposure sets as the number of conditions. For example, imagine a within-subjects design that has two conditions, A and B, and that has four separate assignments with one crossover: half the participants will experience AABB, and the other half will experience BBAA. In the language of repeated measures designs, this experiment has four periods, because there are four different treatment opportunities. However, this experiment only has two different exposure sets, because there are two different sets of assignments corresponding to the two conditions: one exposure set for the first two assignments, and another exposure set for the latter two assignments. Within any exposure set, a researcher can add multiple class assignments (and while it is often desirable to balance the number of treatments in each exposure set, it is also possible for them to be imbalanced in Terracotta), and Terracotta will ensure that the right students see the right versions of each assignment (according to the student's treatment condition in that exposure set).

The advantage of structuring an experiment around an exposure set is that the researcher can specify experimental outcomes at the level of the exposure set, rather than for each period. For example, let us imagine that in the AABB/BBAA experiment above, the first two assignments both focus on mitosis (exposure set 1), and then the subsequent two assignments both focus on meiosis (exposure set 2). To contrast the effect of A and B on students' understanding of these concepts on a later class exam, it should only be necessary to measure students' knowledge separately in each of the two exposure sets. In other words, the researcher should only need to measure two scores (questions about mitosis and questions about meiosis), and it would not be necessary to have one outcome measure for each of the four assignments separately. However, if it is desirable, Terracotta allows many outcome measures to be added to an exposure set.

### Limitations of Terracotta

By manipulating LMS assignments, Terracotta enables experimentation on a wide range of student learning activities and interventions, and these represent important targets for education research. At this time, a Terracotta assignment can contain multiple-choice questions, short answer questions, and file upload response formats, which enables research ranging from well-structured tasks (how students learn science facts) to ill-structured tasks (how students learn literary argumentation; McCarthy et al., 2022). The assignments themselves can contain rich text, links, images, and embedded media. Further, Terracotta enables experimental manipulation of submission policies, grading policies, and feedback policies on these assignments. Nevertheless, Terracotta's scope is limited to the LMS, and this clearly restricts the range of educationally relevant variables that can be manipulated by Terracotta. Additionally, Terracotta's requisite LTI integration with the LMS can also present an obstacle to adoption and recruitment.

LTI tools, like Terracotta, typically require administrative support and endorsement before they can be integrated with a district or institution's LMS—teachers and researchers may request that a tool should be integrated, but the integration is typically approved and managed by administrators. For this reason, schools, districts, and institutions are gatekeepers, and may need to be convinced of the benefits of Terracotta, of embedded experimentation, and of Terracotta's commitment to security and privacy. These are important and beneficial conversations to have, but these may limit the speed or scale with which a researcher might deploy a study. So, while Terracotta automates many of the mechanics of an experimental research study, it still relies on researchers, teachers, and administrators to form partnerships. Once such partnerships are made, however, Terracotta minimizes the effort involved in carrying out a rigorous and responsible experimental research study within a formal education setting.

Unlike laboratory research where participants are typically isolated from one another, student participants in a Terracotta experiment are classmates who are *not* isolated from one another. Students do communicate modestly about schoolwork with their classmates outside of class, commonly by sharing answers, artifacts, and summaries (Asterhan & Bouton, 2017; Bouton et al., 2021). If participants communicate about experimentally manipulated assignments, and this communication exposes them to treatments that were outside their assigned condition, contamination has occurred. Cross-treatment contamination is nothing new in education research (Cook, 2007), and while Terracotta differentiates the treatments that students can access in the LMS, it cannot prevent students from talking with one another. This possibility reinforces the importance of being transparent with student participants: letting them know that, should they agree to participate in a research study within Terracotta, they may have slightly different learning experiences than their classmates, and that they should avoid talking with each other about these experiences. Nevertheless, should cross-treatment contamination occur, this will blur the intended contrast between conditions, and at worst, the consequence would be an underestimate of the effect of an experimental manipulation. In general, researchers should be aware that experimental control is more challenging in the real world, and that there is a risk of observing smaller effect sizes than in the laboratory (Hulleman & Cordray, 2009; Vanhove & Harms, 2015), although sometimes such differences are not observed (Mitchell, 2012).

## Demonstration Study

Terracotta makes it possible to experimentally manipulate consequential aspects of students' educational experiences, to embed complex crossover designs, and to collect streamlined data on these manipulations and their effects. To demonstrate these features, we used Terracotta to conduct a preregistered replication of McDaniel et al. (2012), a well-cited experimental demonstration of the benefits of retrieval practice in a college class using authentic class materials.

In learning contexts, retrieval is the process of accessing knowledge—getting the information *out* of memory—and it is often associated with quizzes or exams. Although these activities are frequently used to measure how much a student has already learned, the retrieval of information on quizzes or exams may also *produce* learning, not just *measure* it (e.g., Roediger & Karpicke, 2006). The act of “getting the information out” requires mental effort and the reconstruction of knowledge, which can lead to robust learning (Roediger & Butler, 2011). Many studies have demonstrated that retrieval practice improves long-term retention, relative to re-reading the same material (Agarwal et al., 2021; Dunlosky et al., 2013; Moreira et al., 2019; Yang et al., 2021). But the McDaniel et al. (2012) study, in particular, had key features that make it ideal for demonstrating Terracotta's capabilities: it manipulated the format of online quiz assignments, it used a within-subjects design, it had repeating treatment periods with multiple crossovers, and it measured the effect of these treatments on students' subsequent exam performance—features that are all supported by Terracotta.

### Method

This demonstration study was approved by the Indiana University Institutional Review Board (IRB) and was publicly preregistered prior to data collection at https://osf.io/juq7n/. All materials, data, and analyses are publicly available at https://osf.io/yrbhe/.

#### Education context

We embedded this study in one section of PSY-P335 Cognitive Psychology during the Fall 2022 academic term at Indiana University Bloomington. P335 is a required course for undergraduate students majoring in psychology. This was an in-person full semester (16-week) section, which had weekly online "reviews" throughout the term, intended to help students learn the material prior to taking in-class exams. The current study was implemented during the second half of the semester (weeks 8 through 15), which is when key features of the current method became available in Terracotta (e.g., cumulative grading for multiple submissions, see *Procedure* below). Total enrollment in the course was 106 students. This course used the Canvas LMS (Instructure, Inc.; Salt Lake City, UT), into which Terracotta was integrated. The instructor of this course is an author of the current study.

#### Participants

Using IRB-approved in-person and email announcements, the instructor invited students to volunteer to participate during the seventh week of the semester. Students were invited to provide consent in an online assignment entitled "Invitation to Participate in a Research Study" (see Fig. 1G), and they were encouraged to complete the assignment within a week; specifically, before the first manipulated review assignment. All students received a small amount of course credit for responding to this assignment prior to the deadline, regardless of whether they chose to provide consent or not.

Ultimately, 77 students submitted responses to this assignment, and among those who provided a response, 39 provided affirmative consent, and these students are considered as participants. Students who did not agree or who did not respond to this assignment were considered nonparticipants, and are excluded from further analysis. The number of students who provided consent was lower than we anticipated in our preregistration. This may have been because consent was administered mid-semester, and students may have been less inclined toward any modification of an established routine. Terracotta does not provide opportunities to directly incentivize students to provide consent (e.g., giving extra credit points only to students who agree to participate), as this would disclose private consent responses to the teacher and possibly create inequities. More research is needed to examine students’ consent decisions and how to increase participation in experimental education research.

#### Materials

The instructor of the course created weekly online reviews for students to revise the course material from a given week. Eight reviews (Reviews 8–15) were included in the current study, and these reviews targeted the following areas of cognitive psychology: Memory (Reviews 8 and 9), Concepts (Reviews 10 and 11), Language (Reviews 11 and 12), Mental Imagery (Review 12), Judgment and Reasoning (Review 13), and Intelligence (Reviews 14 and 15). All reviews contained ten items, with the exception of the last two reviews, which contained six and seven items, respectively.

**Fig. 2:**
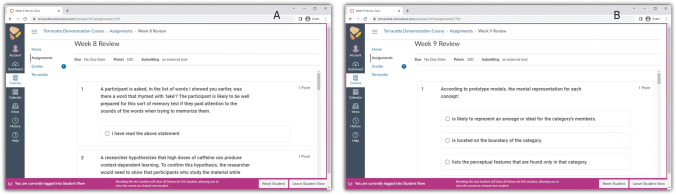
Screenshots of example assignments in Terracotta. The left panel (**A**) shows a Restudy version of the Week 8 Review, and the right panel (**B**) shows a Retrieval Practice version of the Week 9 Review

There were two versions of each review, corresponding to the two conditions: retrieval practice and restudy. If a participant was assigned to the retrieval practice condition on a review, they answered multiple-choice questions; if they were assigned to the restudy condition on the same review, they read correct statements that were the answers to the questions in the retrieval practice version (see Fig. 2).

Three items from each review were selected to appear on the course exam at the end of a unit. Selected items from Reviews 8 and 9 appeared on Exam 2, selected items from Reviews 10–13 appeared on Exam 3, and selected items from Reviews 14 and 15 appeared on Exam 4 (the final exam). Thus, there were a total of 24 multiple-choice questions that were repeated verbatim from retrieval practice versions of the reviews across three course exams. All review items (from retrieval practice and restudy versions both) and a list of those items that appeared on course exams can be found at https://osf.io/yrbhe/.

#### Procedure

During the seventh week of the term, students completed an online assignment that asked for their consent to participate in the current study, and during the eighth week, participants received their first manipulated review (Review 8) as retrieval practice (quiz) or restudy. Participants had a new manipulated review every week until the end of the term, and the format of these alternated on a weekly basis (e.g., retrieval practice one week, restudy the next week, retrieval practice the following week). Students who did not provide consent completed retrieval practice reviews (quizzes) for the remainder of the semester. This was consistent with how reviews were implemented in the first half of the semester (prior to the start of the current study), where all students answered multiple-choice questions on these reviews.

There were eight reviews included in the current study (Reviews 8–15), and each contained ten items, except for Reviews 14 and 15 (which had six and seven items, respectively). Items corresponded to multiple-choice questions in retrieval practice reviews and to correct statements in restudy reviews. Therefore, if participants were assigned a retrieval practice review, they answered multiple-choice questions. If, however, they were assigned a restudy review, they read correct statements that were the answers to the corresponding questions in the retrieval practice review. To ensure their engagement in the task, participants assigned a restudy review checked a box after each statement, verifying that they had read it.

We created two exposure sets on Terracotta with four reviews each: the first exposure set included Reviews 8, 10, 12, and 14, and the second exposure set included Reviews 9, 11, 13, and 15. Participants were assigned to the retrieval practice condition for reviews in one exposure set, and to the restudy condition for reviews in the other exposure set. That is, for roughly half of the participants, the first exposure set made up the retrieval practice condition and the second exposure set made up the restudy condition, and this was reversed for the remaining half of the participants. Thus, all participants were given four retrieval practice reviews and four restudy reviews, and review format alternated on a weekly basis.

Aside from the difference in review format for participants (retrieval practice or restudy), reviews had the same structure for all students enrolled in the course for the duration of the current study. The instructor posted a given week’s review on Canvas each Friday, which students had to complete by class time the following Tuesday^2^. All review items were presented on the same page and in the same order. Students could complete a review up to four times, and they had unlimited time on each attempt. Once they submitted an attempt, students were provided the correct answer and their accuracy on each item (though these were informative only on the retrieval practice reviews). This correct-answer feedback feature, however, was not available on Terracotta until the third week of the current study; instead, students were only provided their accuracy on each item for Reviews 8 and 9. Between attempts, students were required to wait at least two hours to prevent completion of these attempts back-to-back. Students earned 2.5 points each time they completed an attempt, and they could earn a maximum of 10 points if they completed all four attempts^3^. That is, grading of reviews was cumulative based on number of completions, rather than accuracy of responses. The cumulative grading feature became available on Terracotta starting the second week of the current study; until then, students’ grades were adjusted manually to reflect the number of completed attempts. Reviews (including the ones from the first half of the term) made up about 20% of students’ final grades in the course, and all were based on completion (not accuracy).

Over the course of the academic term, students took four exams (three unit exams, and one cumulative final exam), which included some questions repeated from the reviews. The time between a review and a course exam varied, such that the retention interval could have ranged from less than a day (for the last review due prior to an exam) up to 34 days (for the first review following a course exam). Three items from each of Reviews 8–15 appeared on the last three exams. Specifically, items selected from Reviews 8 and 9 appeared on Exam 2, items from Reviews 10–13 appeared on Exam 3, and items from Reviews 14 and 15 appeared on Exam 4 (the final exam). Thus, there were a total of 24 multiple-choice review items repeated on course exams, and scores on these questions were used to compare the mnemonic benefits of retrieval practice and restudy.

Of note, the instructor made all multiple-choice review items (and the answer key to these review items) available to students prior to a course exam. This was done to facilitate exam preparation and to ensure that all students had equitable access regardless of experimental treatment; and the instructor encouraged students to incorporate these practice questions to their study. Put differently, even if participants had experienced some restudy reviews, they still had access to the retrieval practice version before each exam. Considering that this would result in cross-treatment contamination, we expect that this study would yield a more muted estimate of the potential benefits of retrieval practice on memory retention and performance.

Students’ review responses were automatically graded in Terracotta and students’ in-class exam responses were graded using Akindi (a web-based system that automates grading of multiple-choice assessments). After data collection ended, we aggregated scores on the 24 exam questions (those that were repeated from reviews) by student and for each review. Because there were three multiple-choice items repeated from each review, values ranged from 0 to 3, and students who missed a course exam did not have a calculated score corresponding to some of the reviews. We created eight outcomes on Terracotta (four for each exposure set) to manually enter the scores described above. We chose to create an outcome for each review, rather than an outcome for each of the two conditions (retrieval practice and restudy), to allow the analysis of participant behavior across the eight reviews.

#### Statistical analysis

We opted to use Bayesian estimation methods for statistical analyses in the current study. Bayesian estimation provides a framework for making inferences about experimental effects, given observed data and our prior assumptions about these effects. The general advantages of Bayesian inference have been discussed elsewhere (Kruschke, 2011; Vandekerckhove et al., 2018), but the specific benefits for this study include the ability to define a custom analytical model appropriate to the structure of the observed data (e.g., a hierarchical within-subject logistic model) and the ability to deal with unbalanced data (not all students complete all assignments). Also, rather than merely yielding a *p*-value, Bayesian estimation methods produce an informative posterior distribution. The posterior distribution is a direct estimate of the tendency and uncertainty of a parameter in an analytical model, given the observed data and the priors. In this study, despite having knowledge of the expected effect (McDaniel et al., 2012), we elected to use uninformed and vague priors (wide normal distributions centered on zero), so that our replication would provide convincing evidence for skeptical audiences. We characterize the posterior distribution by its modal estimate and by the 95% highest density interval (HDI), which is the range of the most likely parameter values. If the 95% HDI does not include zero or values close to zero, we may infer a credibly nonzero experimental effect.

We sampled the posterior distribution using JAGS (Plummer, 2003) and the runjags package (Denwood, 2016) for R. This was performed using Markov chain Monte Carlo (MCMC) sampling with four independent chains each sampled for at least 30,000 iterations and thinned to every fifth step, following 500 adaptation steps and 1000 burn-in steps. For all parameters of interest, the Gelman–Rubin *R̂* statistic (Gelman & Rubin, 1992) was less than 1.01, and the effective sample size (ESS) was greater than 20,000. Detailed model specifications are available at https://osf.io/b7cwa.

### Results and discussion

#### Treatment characteristics

Each participant was assigned to complete eight different reviews; four were retrieval practice (quizzes) and four were restudy. On average, participants completed 7.74 reviews (*SD* = 0.44); 3.90 were retrieval practice (*SD* = 0.31) and 3.85 were restudy (*SD* = 0.37). Further, participants were incentivized to complete these reviews multiple times with accumulating completion credit up to four submissions. On average, participants made 3.52 submissions to each review (*SD* = 0.43); 3.53 for retrieval practice (*SD* = 0.60), and 3.5 for restudy (*SD* = 0.50). Thus, there was rough equivalence in the number of times participants were exposed to reviews in the two conditions.

However, participants spent more time on reviews when they were in the retrieval practice condition than in the restudy condition. Participants spent an average of 3.71 minutes (*SD* = 2.73) per attempt on retrieval practice reviews, and 1.87 minutes (*SD* = 1.50) per attempt on restudy reviews (bear in mind, however, that attempt duration data have a large positive skew).

In an exploratory analysis, we estimated the parameters of a hierarchical linear model, with the log-transformed duration of each submission as the dependent variable. There were three independent variables: condition (retrieval practice and restudy), assignment (eight different assignments), and submission number (up to four submissions per assignment); coefficients for these variables were estimated for individual subjects and at the group level.

The group-level effect of condition was credibly greater than zero (estimate: 0.35; 95% HDI: 0.25 to 0.44), confirming that participants spent more time on retrieval practice reviews than restudy reviews. McDaniel et al. (2012) did not report time on task; however, laboratory research has similarly observed more time spent completing retrieval practice activities relative to analogous restudy activities when time constraints are not imposed (Üner & Roediger, 2018). We are cautious to interpret the additional time spent on retrieval practice reviews as a source of potential memory benefits, as past laboratory research has shown that additional time spent on a task does not always enhance learning, particularly on rereading tasks (Callender & McDaniel, 2009; Rawson & Kintsch, 2005).

**Fig. 3:**
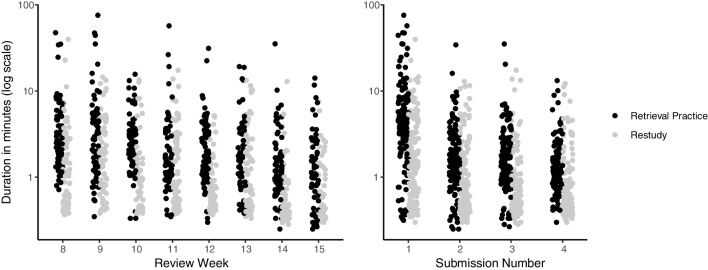
Time spent on reviews. Each dot is an individual student submission, with minor horizontal jitter added. Retrieval practice submissions are shown in black, and restudy submissions are shown in gray. Overall, students spent more time completing retrieval practice reviews than restudy reviews, as is evident in both panels (black dots are higher than grey dots). The left panel shows the duration spent on each individual quiz assignment, with decreasing time across over the semester. The right panel shows the duration spent on each submission, showing decreasing time on each subsequent submission

The effect of assignment on submission duration was credibly lower than zero, indicating that students spent less time on reviews as the semester progressed (estimate: −0.30; 95% HDI: −0.37 to −0.22). And additionally, the effect of submission number was credibly lower than zero, indicating that students tended to spend less time on each subsequent submissions of the same quiz (estimate: −0.32; 95% HDI: −0.40 to −0.24). These effects are shown in Fig. 3.

#### Performance on retrieval practice versions of reviews

Students received completion credit for submitting assigned reviews, regardless of their responses. We did this so that students would receive equitable class credit for completing their reviews, regardless of whether the student had been randomly assigned to restudy (where the only available response was "I have read the above statement") or retrieval practice (which had four different response options, one of which was correct). However, even while students received the same credit for any response, Terracotta still stored students' selections on multiple-choice questions, and it is possible to analyze the accuracy of these responses.

**Fig. 4:**
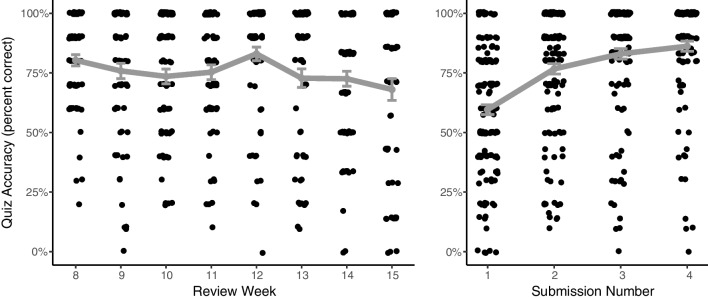
Accuracy on reviews in the retrieval practice condition (quizzes). Each dot is an individual student submission, with minor horizontal jitter added. Gray markers indicate the mean accuracy, and error bars indicate ± one standard error. The left panel shows accuracy on each individual quiz assignment. The right panel shows the accuracy on each submission, with improving accuracy on each subsequent submission

Averaging across all submissions in the retrieval practice condition, participants got 75.4% of items correct (*SD* = 27.0%). To examine how accuracy in the retrieval practice condition changed over the semester, and also how accuracy changed as students made multiple submissions to each assignment, we again conducted an exploratory analysis using a hierarchical linear model, with a logistic response variable (number of correct responses out of number of questions). We found that there was no credible linear change in accuracy over the course of the semester (estimate: −0.07; 95% HDI: −0.25 to 0.11). However, students' accuracy tended to increase in repeated submissions of each review (estimate: 0.88; 95% HDI: 0.69 to 1.08), suggesting that students in the retrieval practice condition were using the reviews as an opportunity to learn and improve, despite receiving full credit for any submission. These data are shown in Fig. 4.

#### Learning outcomes

**Fig. 5:**
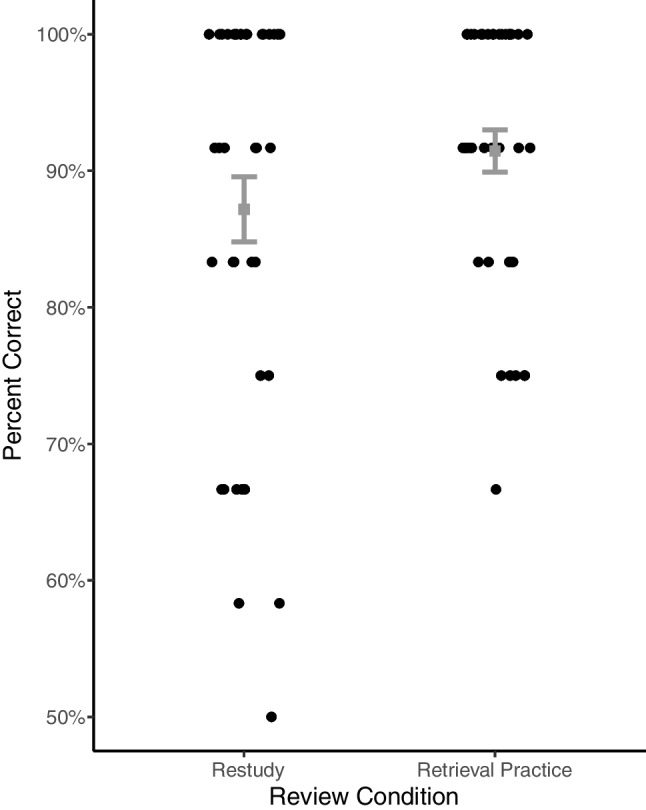
Percent correct on subsequent exams. Each dot is an individual student, showing the percentage of items the student got correct on each set of questions associated with reviews. Minor horizontal jitter added to show density. Gray markers indicate the mean percent of items answered correctly for all participants, and error bars indicate ± one standard error

Three questions from each individual review were included in subsequent in-class exams. Student's scores on these questions, specific to their associated review condition (retrieval practice or restudy), were manually added to Terracotta by the instructor. Overall, participants performed well on these questions, getting an average of 2.68 correct out of 3 (89.3%; *SD* = 0.32). However, students were more likely to answer questions correctly if they had previously been included in retrieval practice reviews (2.74 correct; 91.5%; *SD* = 0.29), compared with questions previously included in restudy reviews as correct statements (2.62 correct; 87.2%; *SD* = 0.45), and our preregistered analysis estimated this difference between retrieval practice and restudy conditions to be credibly greater than zero (estimate: 0.51; 95% HDI: 0.015 to 1.05), as shown in Fig. 5.

The 4% difference we observed is smaller than the roughly 10% difference between restudy and retrieval practice reported by McDaniel et al. (2012). This may be because students in this section of Cognitive Psychology were informed about the benefits of retrieval practice, and the instructor made review questions (along with their answers) available so that students could use retrieval practice for exam preparation. Participating students were also aware that the review format was manipulated; thus, it is possible that they engaged in self-testing even when assigned to the restudy condition on half of the reviews. Furthermore, the reduced effect size in the current study may also be due to differences in the scope of the studies' implementations, as the current study manipulated eight quizzes and measured outcomes on 24 exam questions, whereas the original study manipulated 15 quizzes and measured 84 exam questions. Furthermore, given the low consent rate observed in the current study (~37%), sample bias and ceiling effects may be affecting these estimates (exam scores in the current study are higher than in McDaniel et al., 2012). Despite these considerations, however, a 4% difference in performance on exam questions is non-negligible, and demonstrates that manipulations within Terracotta can have meaningful consequences for student learning.

### Summary of demonstration study

Successfully replicating McDaniel et al. (2012), we observed a credible improvement in exam performance in an authentic education setting, after participants had practiced retrieving the material (retrieval practice) compared with rereading the material (restudy). This experimental manipulation took place entirely within Terracotta, after students were informed about the research in full transparency and given agency in deciding whether to participate.

We do not claim that this demonstration study is emblematic of all research that could be conducted within Terracotta. Terracotta's feature set enables a wide range of possible experimental manipulations and designs, so that any single study would be unrepresentative on its own. Nevertheless, beyond its successful replication, two things are particularly noteworthy about this demonstration: (1) the ease with which we were able to embed this experiment into the class, and (2) Terracotta's ability to automatically collect granular data on student behaviors when interacting with manipulated assignments. As to the ease of embedding, we estimate that experiment setup took a total of 2 minutes, assignment construction took roughly 10 minutes per assignment, and manual entry of outcomes took about an hour. A video of the setup and creation of two assignments is available at https://osf.io/24qp7. Anecdotally, for an embedded experiment with informed consent, a within-subject design, eight treatments, alternating crossovers, and exam scores mapped to treatments, this represents a dramatic time savings.

As to Terracotta's granular data collection, the current study extends past research conducted in laboratory settings (Üner & Roediger, 2018), observing in particular that participants in authentic education settings spend more time performing retrieval practice activities compared with restudy activities. This is noteworthy because, in the current study, participants in the restudy condition had academic incentives to learn the material, which is not necessarily the case in the lab. Indeed, supplemental reading in authentic education settings is correlated with improved performance (Carvalho et al., 2018). Nevertheless, the current study demonstrates that *assignments* to restudy result in quantitively less time on task, and less educational benefit, than assignments to practice retrieving the material. Analyses of time spent on self-regulated learning activities are potentially fruitful avenues for future research (Carvalho et al., 2022; Knight et al., 2017; Son & Kornell, 2009).

We are careful to label this a *demonstration* study and not a *validation* study. As with any behavioral research tool, measurement validity in Terracotta is principally determined by how it is used and is not an invariant property of the tool itself. A hammer might be a valid means for driving a nail, but not for turning a screw—and similarly, not all education research studies are suitable for implementation in Terracotta, or in any single tool. Nevertheless, the current study demonstrates that Terracotta can be used to experimentally manipulate consequential aspects of students' experiences in education settings, to embed complex crossover designs, and to collect streamlined data on these manipulations and their effects.

## Conclusion

Terracotta enables a wide range of experimental research manipulations and designs, which aim to match the wide range of instructional decisions and interventions that might be implemented with LMS assignments. We have demonstrated how Terracotta might be used to easily test a cognitive manipulation of LMS assignments, but there are other research approaches in education that can also benefit from having Terracotta in their methodological toolkits. For example, Terracotta might also be used to test social and motivational interventions, and to test the effectiveness of learning resources and instructional strategies that are presented to students in assignments. Moreover, by eliminating many of the difficulties of implementing an experiment in a single class, we further hope that Terracotta might be used to deploy experiments that are distributed across many classes, thus building our understanding not only of what works in education, but also *where* it works (Churches et al., 2020; de Leeuw et al., 2022; Fyfe et al., 2021).

Terracotta is a research tool and is not intended for routine practice—which means that once an effective learning strategy is identified within Terracotta, it will require additional work to disseminate this finding and to improve practice more broadly. How research findings can be used to affect routine practice is a steep challenge in education (and in all disciplines) that is unlikely to be addressed by any single method or tool. Nevertheless, Terracotta might make inroads. By lowering the barriers to experimental research in authentic class settings, and by involving teachers in the practice of experimental research, the distance segregating research findings from education practice may be reduced (Mace & Critchfield, 2013; National Research Council, 1999).

Terracotta's specific goal is to lower the practical barriers to easy, accessible, responsible, and rigorous experimental research across education levels, student populations, and learning materials. By allowing researchers to embed studies in the LMS, where many learning activities already take place, Terracotta can help advance our understanding of what works in student learning on a broad scale and help build stronger evidence of how to improve education.

## Learning more

This article has stated a general need for experimental research infrastructure in education, given a broad overview of how Terracotta addresses this need, and described the specific methods and results from an authentic research study using Terracotta. To learn more about Terracotta, and how one might get Terracotta integrated with the LMS at a specific school, we encourage readers to visit the Terracotta website at https://terracotta.education. Presently, you will find a frequently-asked-questions (FAQ) page, a “quick start” guide showing each step in the process of creating a simple experiment, and a form for contacting us and getting support. We expect this documentation to grow and evolve over time.

## Footnotes

1 We acknowledge, however, that a teacher may infer whether students provide consent to participate and their condition assignments when grading or reviewing student submissions in Terracotta. This is because individual student submissions will differ according to different experimental treatments. If this creates potential issues, we recommend that a teaching assistant (who is not involved in the research), might be responsible for manual grading or reviewing submissions.

2 Late submissions were not accepted on reviews; however, students could request up to two 24-hour extensions on each review. Though this would have extended the completion window of these assignments, the instructor received only two extension requests over the course of the study. Additionally, the completion window of reviews was narrower for the first half of the semester (reviews due Monday rather than Tuesday), and this was changed prior to the start of the current study based on student feedback.

3 During data collection, we identified and resolved minor bugs in Terracotta that prevented some students from successfully submitting their review and earning credit for these submissions. This happened to a small number of students during the first two weeks of the current study (some of these students could have been nonparticipants), and the students experiencing these issues were given full credit on those reviews.
